# Analyzing the Potential of Laser Femtosecond Technology for the Mass Production of Cyclic Olefin Copolymer Microfluidic Devices for Biomedical Applications

**DOI:** 10.3390/polym17091289

**Published:** 2025-05-07

**Authors:** Irene Varela Leniz, Taieb Bakouche, Malen Astigarraga, Florent Husson, Ane Miren Zaldua, Laura Gemini, José Luis Vilas-Vilela, Leire Etxeberria

**Affiliations:** 1 Leartiker S. Coop, Xemein Etorbidea 12A, 48270 Markina-Xemein, Spain; 2 ALPhANOV, Institut d’optique d’Aquitaine, Rue François Mitterrand, 33400 Talence, France; 3 Macromolecular Chemistry Research Group (Labquimac), Department of Physical Chemistry, Faculty of Science and Technology, University of the Basque Country (UPV/EHU), 48940 Leioa, Spain; 4 BC Materials, Basque Center for Materials, Applications and Nanostructures, UPV/EHU Science Park, 48940 Leioa, Spain

**Keywords:** microfluidics, thermoplastics, laser processing, injection molding, surface analysis, polymer bonding

## Abstract

Precision micromilling is currently widely used for the fabrication of injection mold inserts for the mass production of microfluidic devices. However, for complex devices with micrometer-scale and high density of structures, micromilling results in high production times and costs for production runs of hundreds or thousands of units. Femtosecond laser (fs-laser) technology has emerged as a promising solution for high-precision micromachining. This study analyzes the potential of fs-laser micromachining for the fabrication of injection mold inserts for the large-scale production of thermoplastic microfluidic devices. For the evaluation of technology, a reference design was defined. The parameters of the fs-laser process were optimized to achieve high resolution of the structures and optimal surface quality, aiming to minimize production times and costs while ensuring the quality of the final part. The microstructures were replicated in two different grades of COC (Cyclic Olefin Copolymer) by injection molding. The dimensional tolerance of the structures and the surface finish achieved both in the insert and the polymer parts were characterized by scanning electron microscopy (SEM) and confocal microscopy. The surface quality of the final parts and its suitability for microfluidic fabrication were also assessed performing chemical bonding tests. The fs-laser machining process has shown great potential for the mass production of microfluidic devices. The developed process has enabled for a reduction of up to 90% in the fabrication times of the insert compared to micromilling. The parts exhibited very smooth surfaces, with roughness values (Sa) of 64.6 nm for the metallic insert and 71.8 nm and 72.9 nm for the COC E-140 and 8007S-04 replicas, respectively. The dimensional tolerance and the surface quality need to be improved to be competitive with the finishes achieved with precision micromilling. Nonetheless, there is still room for improvement considering the significant reduction in the production times through new laser processing strategies.

## 1. Introduction

The interest in microfluidic devices has grown rapidly in recent years due to their high potential in multiple cutting-edge applications. The main competitive advantages that the technology offers are the reduction in reagent volumes, reduced sample processing times and streamlining of lab procedures [1]. Microfluidic devices constitute a groundbreaking advancement in biotechnology and biomedical applications: in point-of-care diagnostics, they offer rapid and high-sensitivity detection of pathogens and biomarkers with minimal sample volumes; in bioassays, they facilitate high-throughput screening or enable controlled cell culture environments; lab-on-chip platforms combine various laboratory procedures onto a single microfluidic device, enabling miniaturized and automated workflows; lastly, microfluidic platforms have revolutionized tissue engineering and organ-on-chip technologies, mimicking the in vivo microenvironment of human tissues [2,3,4,5,6,7]. But the scale-up of microfluidic prototypes to marketplace still experiences limitations due to the difficulties involved in their mass production. These are mainly attributed to the strict tolerances that devices must meet through cost-effective processes [8].

With the growing demand for mass production, traditionally employed materials in microfluidics industry, such as silicon or glass, have been left behind by low-cost polymers. Polystyrene (PS), polycarbonate (PC), polymethyl methacrylate (PMMA), or cyclic olefin copolymer (COC) are some examples of the mostly employed polymers due to their biocompatibility, chemical resistance, optical transparency and ease of transforming [9]. Among them, the use of COC material is widely extended for microfluidic applications due to its low autofluorescence, strong chemical resistance and low drug absorption, and it is also reported to be a cost-effective material for the mass production of microfluidic devices [10,11]. Several works are reported in the literature employing COC material for microfluidic point-of-care, lab-on-chip or organ-on-chip applications [11,12,13,14,15,16].

Polymeric devices produced from a mold guarantee fabrication consistency, with a high efficiency and a low cost per unit [8]. In this regard, polymer injection molding is a well-established technology for industrial-scale production, due to its high stability, replication, high throughput and cost-effectiveness [17,18,19]. Furthermore, it is a unique mass production-compatible technology capable of creating complex 3D geometries for microfluidic devices. Nonetheless, high cost related to the mold is a major drawback of microfluidic injection molding [8].

The large-scale production of polymeric microfluidic devices typically involves the fabrication of a master mold, the replication of the mold structures in low-cost materials and back-end processes, such as bonding, assembly of components or surface treatments [8].

Microfluidic molds are typically composed of two main parts with features of different dimensions: the master mold and the insert. The master mold provides structural support and interfacing with the external world and is usually fabricated by conventional machining. Instead, the insert includes the micro- and/or nanoscale features, which provide specific functionality to the device, and thus their cost is relatively high compared to the master mold. There are usually multiple interchangeable inserts for a unique master mold, which contributes to reduce the cost of the mold and increases the flexibility of the technology. In the case of design change in the microfluidic features, it will only be necessary to re-fabricate the insert and not the entire mold [18,20]. In this work, we focus on the fabrication of the insert, as it is the accuracy and surface quality of the insert features which determine the performance of the microfluidic device and final device cost.

Micromilling, laser ablation, electrical discharge machining or *LIGA* (the process of combining lithography, electroplating, and molding) are commonly employed fabrication techniques of microfluidic molds [21]. Among them, micromilling is widely used, as it can produce 3D microstructures with high efficiency and good surface quality [21,22]. However, overall cost including milling tools and machining time can be high [8]. Such high costs can hardly be justified in small-volume productions (<1000–10,000 units) and when the device’s final cost is decisive.

Micromachining by fs-laser is a well-known manufacturing technique which enables obtaining high-quality machined parts with micrometric-resolution features thanks to its intrinsic properties of minimizing thermal effects linked to the laser–material interaction [23]. Moreover, the current industrial availability of high-power fs-laser sources grants the possibility to achieve shorter processing times and overall competitive process throughputs with respect to more conventional manufacturing methods [24].

In some previous studies, laser micromachining and thermoplastic injection molding technologies have been combined for the fabrication of microfluidic devices. Trotta et al. [25] presented a PMMA device for optical manipulation of single cells produced from a microstructured injection-molded insert. Fs-laser milling and μEDM machining were employed in this study for the fabrication of the microfeatures on the insert. In another study, Saadat et al. [26] demonstrated a rapid prototyping technique using fs-laser to create metallic molds for PDMS microfluidic devices. The devices produced showed very smooth surface, which improved the fluid flow through the channels. They suggest some optimizations in the laser processing of the mold to minimize the tapering of the channel walls.

No work was found in the literature employing any grade of the COC material, which is considered a reference when it comes to materials for microfluidic device fabrication [27]. No cost-effectiveness study of the process combining laser micromachining of mold inserts followed by thermoplastic injection molding was found either; instead, previous studies mainly focused on validating the feasibility of the process.

In this work, we aimed to assess the potential of the fs-laser micromachining technology for the mass production of microfluidic devices for biomedical applications, as a cost-effective solution for the fabrication of injection molding inserts. Two different COC grades (an amorphous and a semicrystalline grade) were selected to replicate the features machined in the inserts. Validation of the machined insert and of the injection-molded polymer parts were performed by (1) characterizing the structures by scanning electron microscopy (SEM), (2) measuring structures dimensions and roughness by confocal microscopy, and (3) assessing the compatibility of the polymer parts with a microfluidic bonding process.

## 2. Materials and Methods

### 2.1. Microfluidic Design

**Figure 1:**
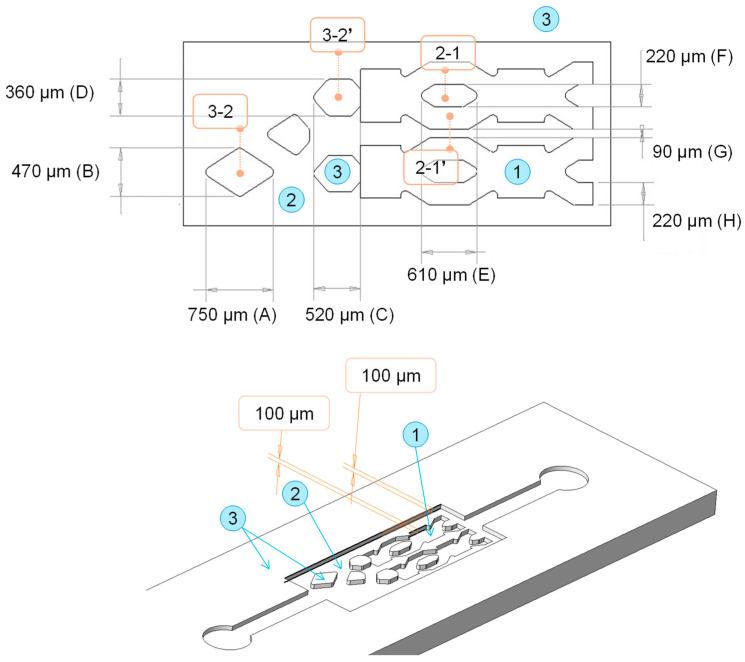
Microfluidic reference design. At the top, 2D design indicating the width structures (x, y dimensions) from A to H, and at the bottom, 3D design of the polymer replica indicating the structures. The surfaces with different finishes according to the height of each structure are categorized with numbers from 1 to 3.

A reference design was created using CREO Parametric (9.0.2.0) software (PTC Inc., Boston, MA, USA). This was a non-functional design that included various microstructures in the shape of fluidic channels and chambers common in microfluidics. The term “non-functional” refers to the fact that with the final part obtained, it will not be possible to generate a chip that will enables a microfluidic assay to be performed, but rather it will be a design for the evaluation of the fabrication technology. The width (dimensions *x*, *y*) and the height (dimension *z*) of the structures are indicated in Figure 1. The surfaces at different heights are categorized with numbers from 1 to 3. Surface 1 is the initial surface of the insert that was not laser machined, and its quality corresponds to the insert substrate fabrication process. Surfaces 2 and 3 have the finish corresponding to laser machining and are located at 100 and 200 µm height, respectively, from the initial surface.

### 2.2. Metallic Mold Insert Fabrication

1.2083 ESU stainless steel (Meusburger GmbH, Viernheim, Germany) was selected for the manufacture of metallic inserts. This is highly recommended for medical and optical products because of its suitability for mechanical polishing and high-quality finishes [28].

**Figure 2:**
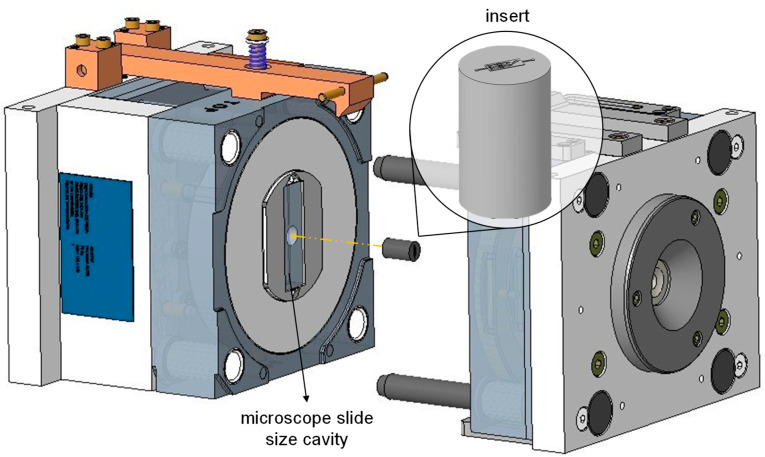
Schematic diagram of the main injection mold and insert assembly.

The insert substrate was milled so that it could fit a reconfigurable injection mold owned by Leartiker (see Figure 2). The surface to be engraved was then grinded by an abrasive diamond well to improve its surface finish after milling.

### 2.3. Manufacturing of Structured Microfluidic Molds by Fs-Laser Processing

A Satsuma X laser system (Amplitude Laser, Pessac, France) running at a 1030 nm central wavelength and a pulse duration < 450 fs, equipped with pulse-on-demand and GHz-burst options, was employed for all tests. The laser beam was shaped and injected into a galvanometric scanning system ExcelliSCAN20 (SCANLAB GmbH, Munich, Germany) to precisely deflect the beam according to the designed geometry. The laser beam was focused onto the insert surface by a 100 mm focal length f-theta lens, so as to achieve a focused laser sport diameter of approximately 31 µm.

Several combinations of laser parameters and scanning strategies were tested to find the optimal parameters in order to meet the target dimensions of the structures presented in Figure 1. Two main limitations were addressed during the testing phase: first, the engraving quality and tapering effect at the edges of the structures (over-engraving issues), and second, the final surface roughness of the overall insert after laser processing.

Firstly, different scanning approaches were tested to minimize the over-engraving at the edges of the structures. This optimization was critical to the functionality of the final microfluidic device. Poor quality at the edges of structures can cause issues in subsequent bonding and assembly processes of the final microfluidic polymer parts. Over-engraving effects occur due to mechanical inertia of the scanning system and the intrinsic shape of the Gaussian laser beam energy profile. Indeed, the acceleration and deceleration of the deflecting mirrors during laser processing led to higher laser-spot spatial overlapping at the beginning and at the end of the scanning trajectory, just at the edges of the structures, which in turns lead to more important ablation effects. To bypass this issue, the pulse-on-demand option offered by the laser system can be employed [18]. This option automatically modifies the laser repetition rate for a given scanning speed and energy per pulse, and it compensates for the acceleration/deceleration dynamics of the scanner mirrors.

On the other hand, the tapering effects linked to the use of a laser beam with Gaussian-shaped energy distribution are intrinsically always present in laser-based processes. In this case, to address the issue, an additional scanning strategy was employed for the manufacturing of the final insert: to generate a chamfer at the edges of the treated regions by progressively decreasing the extension of the region to be treated.

Despite the fact that the selected approaches do not guarantee the shortest processing time, they enable finding a working compromise between the final wall tapering and the engraving quality on the edges of the structures.

Secondly, with respect to the final surface roughness, it is well known that the surface quality of a laser-engraved material is strictly linked to the processing wavelength, among other parameters. As a rule of thumb, the shorter the laser wavelength, the better the surface quality at the bottom of the engraved area [29,30]. Nonetheless, it is necessary to exploit the highest average power available from the laser source to obtain high enough ablation rates and minimize the insert processing times as possible. Therefore, in this work, a two-step processing method was employed to achieve the lowest surface roughness on the insert while maintaining the processing time within competitive values by operating the laser source at 1030 nm. First, the geometry was engraved on the insert. In a second time, by employing the same processing setup and optical configuration but modifying the processing parameters only, a polishing step was carried out to minimize the final surface roughness. Surface polishing by fs-laser is a fairly new technique that enables obtaining low-roughness surfaces on metals with very high spatial resolution and negligible impact on the material bulk properties [31]. Laser polishing techniques are based on the possibility to form a melting pool on the material’s surface after thermal accumulation due to deposition and absorption of the laser energy.

The final processing parameters employed for the engraving and polishing step are summarized in Table 1.

### 2.4. Replication on Polymers by Injection Molding

Two different grades of Topas^®^ COC were selected for replication of the structures in polymers: medical-grade semicrystalline COC E-140 and medical-grade amorphous COC 8007S-04, with a density of 0.94 g/cm^3^ and 1.01 g/cm^3^, respectively [32,33]. Both materials were purchased from TOPAS Advanced Polymers (Oberhausen, Germany).

Several studies have shown differences in the replicability of micro-scale geometries according to the different polymer properties, as melt viscosity or thermal properties [34]. In this sense, the differences in macromolecular structure and thermomechanical behavior have been analyzed in our previous work [35], which enabled determining the norbornene content and the thermal transitions of both COC grades.

Replicas in COC E-140 and 8007S-04 were obtained by injection molding in Arburg Allrounder 270A 250-70 machine (Arburg GmbH, Lossburg, Germany) with a clamping force of 25 Ton, a screw of 20 mm diameter and a maximum injection volume of 80 cm^3^. The injection molding process was performed within ISO7 cleanroom facilities at Leartiker S. Coop.

According to the mold configuration shown in Figure 2, cylindrical inserts were assembled into another interchangeable insert of the main mold owned by Leartiker S. Coop. The interchangeable insert generates a cavity with dimensions that correspond to those of a standard microscope slide. As a result, the final injection-molded polymer parts were of slide size, with the microstructures patterned on one of its sides. No release agents were applied in the mold either so as not to alter the superficial chemistry of the injected polymeric parts.

Table 2 shows the optimized process parameters used during injection molding to fabricate the polymer replicas in the two different COC grades. The selection of the parameters is based on the specific properties of the two material grades, as well as the need to produce high-quality parts.

### 2.5. Dimensional Characterization of Microstructures and Surface Roughness Analysis

Dimensional control and surface finish analysis on the insert and polymer parts were performed using a confocal microscope for materials (Zeiss LSM 900, Carl Zeiss Microscopy GmbH, Jena, Germany). For the dimensional measurements of the structures, the EC EPIPlan 10×/0.25 objective from Zeiss was used. For the surface roughness analysis, topography images were acquired using the objective C Epiplan-APOCHROMAT 50× from Zeiss. ConfoMap^®^ V10 surface analysis software was used to process the images acquired by the confocal microscope and structures dimensions measurements and surface roughness analysis. A scanning electron microscope (SEM VEGA3, Tescan, Brno, Czech Republic) was employed for all observation.

### 2.6. Sealing of the Structures by Chemically Assisted Bonding

**Figure 3:**
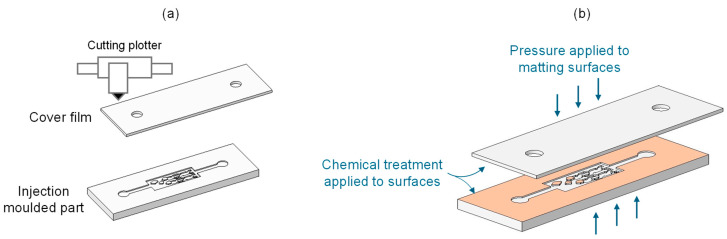
(**a**) Manufacturing of the parts that make up the assembly. The upper part is a cover film made using a cutting plotter. The lower part is the injected piece with the microstructures. (**b**) Schematic of the bonding process employed.

A room-temperature surface treatment was applied for sealing the structures on the polymer replicas with a cover of COC E-140. A schematic of the bonding process is shown in Figure 3. For the fabrication of the cover, a cutting plotter (Summa S2 T75, Summa, Gistel, Belgium) was used to cut a 100-micron sheet of COC E-140 that matched the dimensions of the injection-molded polymer replicas. The liquid inlet and outlet ports were also cut to match the circular ports of the replicas.

The low-temperature and low-pressure chemically assisted bonding approach employed avoided any structure distortion during bonding. (3-aminopropyl)triethoxysilane (APTES) silane reagent was used to modify the surface of the parts and produce an irreversible bond between them. First, surfaces were treated with corona discharge and then immersed in APTES aqueous solution for a defined time. The matting surfaces were then dried with air and brought into contact for a defined time. Further details on the process parameters cannot be disclosed, as it is protected by Leartiker S. Coop as a trade secret under the registration number 2403017196786 in Safe Creative on 1 March 2024. Bonding coverage was then assessed using a Nikon Eclipse 80i microscope (Nikon Corporation, Tokyo, Japan).

## 3. Results and Discussion

### 3.1. Fabrication Time and Cost

A total of 45 min was required for the engraving and polishing of the microfluidic design using the fs-laser. Considering the time and equipment used, it has been estimated that the manufacturing cost of the insert would be in the range of EUR 500–EUR 1000.

### 3.2. Dimensional Characterization of the Fabricated Microstructures

**Figure 4:**
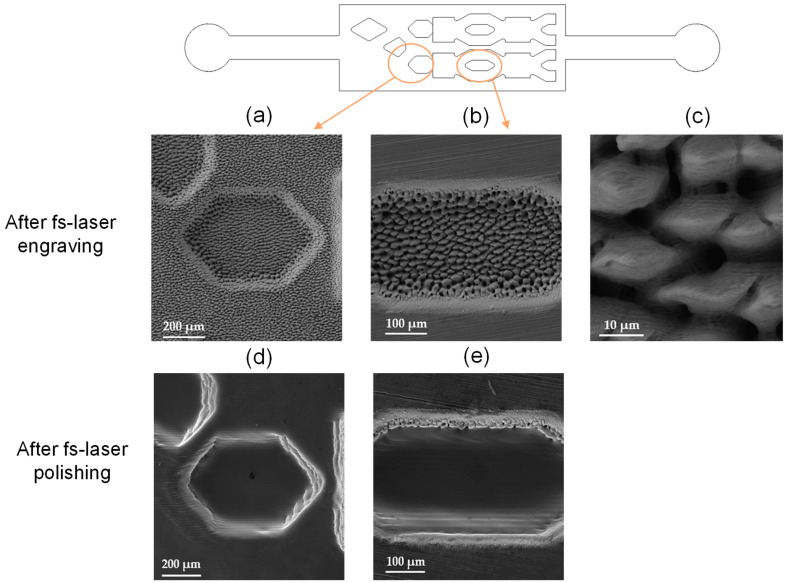
SEM images of the fs-laser-engraved insert (**a**,**b**,**d**) and the same insert after fs-laser polishing (**c**,**e**).

SEM images of the surface quality after fs-laser engraving and fs-laser polishing are presented in Figure 4. Figure 4c is representative of the surface at the bottom of the engraved areas at the two different engraving depths shown in Figure 1; Figure 4a at 100 µm and Figure 4b at 200 µm, categorized as surfaces 2 and 3, respectively. The formation of spike structures with a width of up to 20 µm is easily visible. These structures generally form when high energy doses are delivered to the material surfaces because of the onset of hydrodynamic effects caused by thermal accumulation and incubation [36]. Figure 4d,e present the same engraved areas after fs-laser polishing. Large periodicity waviness is visible on the surface after laser polishing. The appearance of this feature is to be attributed to the non-flat Gaussian energy distribution of the laser pulses and can be optimized by employing a larger laser-focused spot where the energy distribution gradient is less steep.

Table 3 presents the height of the microfluidic structures defined in the different zones of the benchmark design (see Figure 1). Theoretical dimensions, measurements taken on the insert and measurements on the COC E-140 and 8007S-04 injection-molded replicas were compared. For the characterization of the replicas, n = 3 parts were measured for each COC grade and the mean and standard deviation values were computed per measuring zone. The error of the insert with respect to the theoretical dimensions defined by design was calculated. Instead, considering the error measured in the polymer parts is the sum of laser processing error and injection molding replication error, in order to determine the error originating only from the replication process, dimensions in the polymer parts were compared with those on the insert.

As for the insert, the error in the channel height measurements with respect to the designed dimensions was in all cases below the threshold of 10% defined for this analysis. The same tolerance has also been applied in other studies by other authors assessing microfluidic device fabrication processes [37]. In the case of the injected polymeric parts, a greater deviation was measured compared to the dimensions in the insert, particularly for the structures in zones E-F and G. This same area exhibited the highest error values in the measurements taken on the insert. In all cases, the measured height of the structures was below 100 microns, indicating that the polymer melt during the injection molding process was unable to completely fill the machined cavities in the insert. High-fidelity replication is more challenging in cavities with a higher aspect ratio and/or in areas with a high density of structures, such as zones E-F and G, compared to zone A-B with a lower density of structures and aspect ratio, where the polymer melt flow could more easily fill cavities.

**Figure 5:**
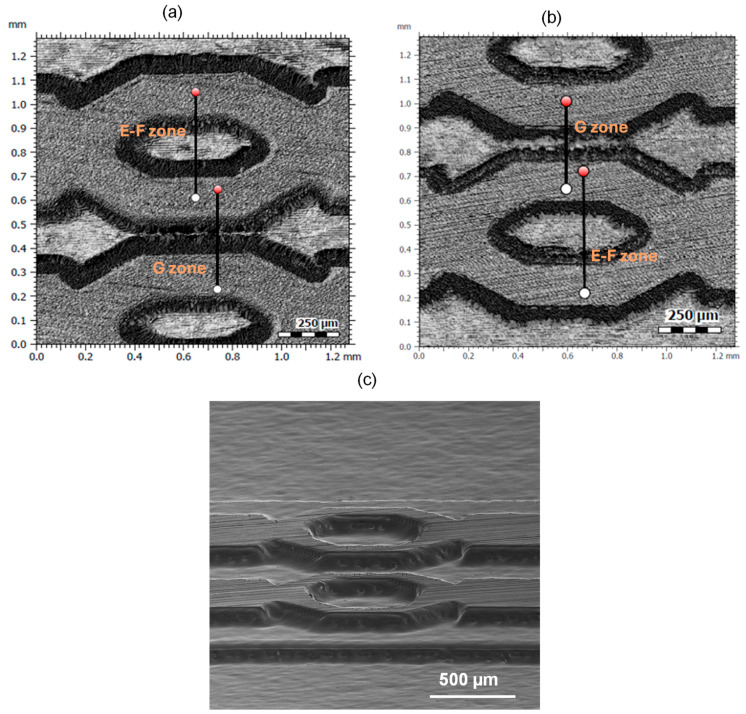
Confocal microscope images of the insert (**a**) and polymer replica (**b**) in E-140-grade showing E-F and G zones. (**c**) SEM image of the E-140-grade replica.

Channel width characterization was not as straightforward due to the over-engraving effect at the edges of the structures originated during the laser manufacturing of the insert. The laser penetrated deeper than intended, removing more material than desired at these areas and leading to inaccuracies. The over-engraving of the borders in both the insert and the polymer replica can be seen in Figure 5 as black areas around the edges with an approximate thickness of between 50 and 150 microns.

**Figure 6:**
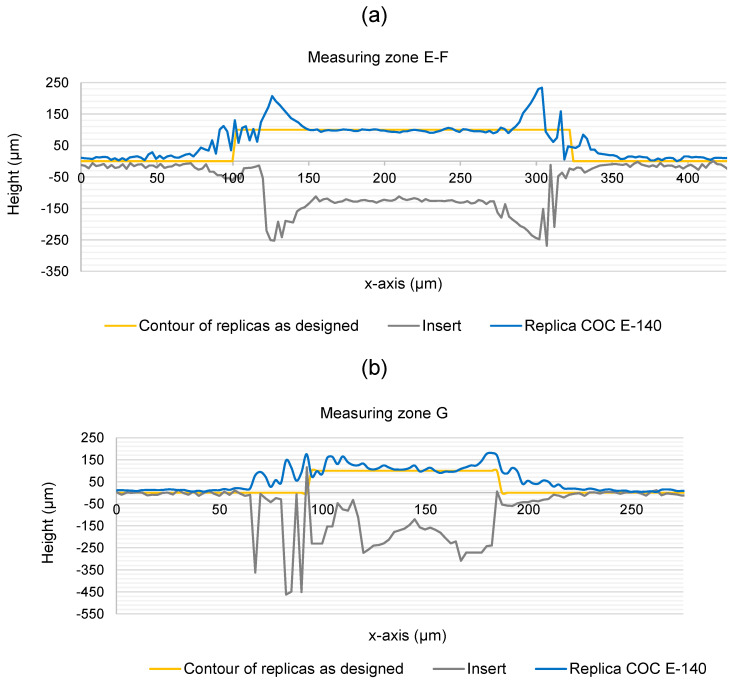
Profiles of the structures in the zones E-F (**a**) and G (**b**) along the section lines indicated in Figure 5a,b.

Capturing vertical walls using the confocal microscope is in itself challenging since the technique relies on reflected light, but the irregularities at the edges due to the over-engraving also generated noise in the acquired images. This can be observed in the profiles of the structures in Figure 6. It was not possible to generate structures with profiles with zero taper walls in the insert using laser. Instead, rounded edges with defects in the form of peaks and valleys were obtained. Part of these defects are also believed to be burrs and material redepositions from the laser machining process. These defects were transferred with the same shape to the polymer replicas after the injection process as shown in Figure 6a (E-F zone) and Figure 6b (G).

Therefore, reliably determining where the edge of the structure is located was more difficult, as it highly depended on the user computing the measurement. As a criterion for this analysis, the outer contour of the over-engraved edge was established as the edge of the structures.

Table 4 presents the widths of the structures measured in the insert and the COC E-140 and 8007S-04 replicas. As in the height analysis, the error in the insert with respect to the designed dimensions and the error in the polymer replicas with respect to the insert were calculated. A maximum error of 39% was measured in zone G in the insert. The height analysis results presented above also showed that this zone is the most challenging to machine and replicate due to having a higher aspect ratio than the others and being located in an area with a higher density of structures. Additionally, in all cases, the width values in the insert were greater than the designed dimensions in all zones. This was an expected result considering that according to the criterion followed, the edge of the structures was defined as the outer contour of the over-engraved area. The actual edge should be somewhere around the middle of the total width of the over-engraved area. Instead, the measurements in the COC parts have shown high replicability with an error of less than 2% in the less dense areas of the structures (zones A to D). The zone that recorded the highest error in both COC grades was again zone G.

### 3.3. Analysis of the Surface Roughness

Figure 6 shows the topographic images of the different surface finish present on the polymer replicas according to the labelling defined in Figure 1. On the one hand, the initial surface, prior to laser processing, originates from the grinding process used in the manufacturing of the insert. This corresponds to surface 1, which is the upper surface or the highest structures of the insert. On the other hand, surfaces 2 and 3 were processed by laser according to the benchmark design, and their finish corresponds to laser polishing.

**Figure 7:**
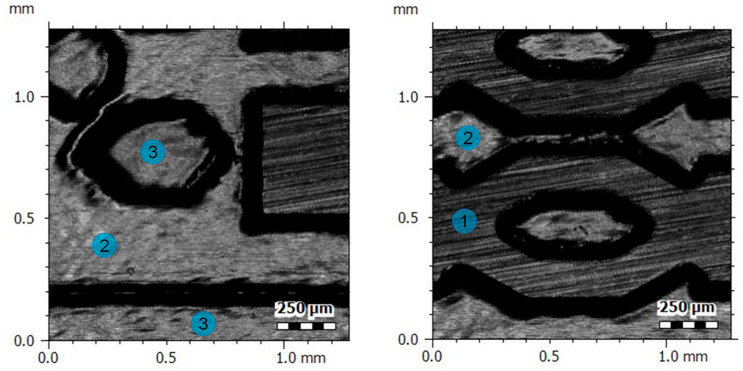
Confocal images showing different surface finishes in a COC E-140-grade polymer replica. Surface types from 1 to 3 are numbered according to the references defined in Figure 1.

**Figure 8:**
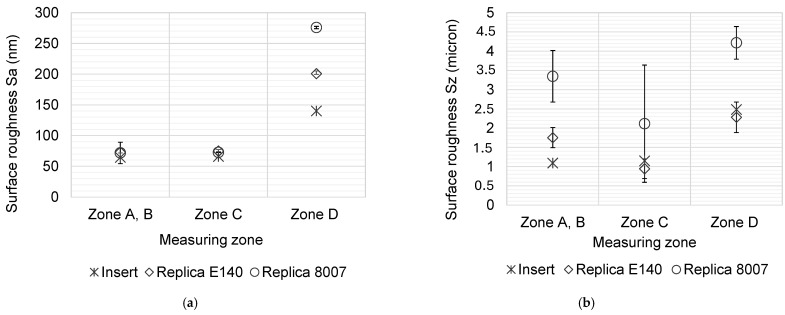
Surface roughness Sa (**a**) and Sz (**b**) values at different zones of the design and for different substrate materials.

In Figure 8, the mean values and standard deviations obtained for three average surface roughness (Sa) and maximum surface roughness (Sz) measurements taken on the insert and the replicas in the two grades of COC are compared. A consistent trend is observed in both Sa and Sz measures, where the insert shows the lowest roughness, followed by the COC E-140 replicas, and finally the COC 8007S-04 replicas with the highest roughness. This consistency suggests that the surface characteristics for the three different sample types remain uniform regardless of the roughness parameter considered. The differences related to the effect of the manufacturing process are also notable. The regions processed by laser (surfaces 2 and 3 in Figure 7) showed lower surface roughness compared to initial grinded surface (surface 1 in Figure 7), indicating that the femtosecond laser provided a smoother and more uniform surfaces, which is directly related to lower Sa and Sz values, respectively. No significant differences were identified for surfaces at different heights, with Sa values of 65.9 nm and 64.6 nm for surfaces 2 and 3 measured at the insert, respectively. Comparing these results with the Sa value of 140.2 nm measured on surface 1, laser polishing has managed to reduce the initial roughness of the grinded insert by up to 53.9%. Regarding the polymer replicas, Sa values of 71.8 nm and 72.9 nm were measured for COC E-140 and COC 8007S-04, respectively, both on surface 3. The Sa roughness values obtained in this work represent an improvement compared to similar results in the state of the art [26,38] in other polymer materials, even though no studies have been found using any grade of COC.

The standard deviation of the average Sz values for each sample type in Figure 8b is high, which could indicate a lack of reliability in the results. However, it should be considered that Sz is more sensitive to imperfections and defects on the surface, as any extreme peak or valley will significantly affect the measure. This can increase data dispersion, and therefore the standard deviation. Again, higher Sz values are observed for the COC 8007S-04 replicas compared to COC E-140 replicas.

The differences in material properties, as well as the defined injection molding parameters, are believed to result in differences in the measured roughness values of the two grades of COC. However, observing the Sa values for laser-polished surfaces (2 and 3), there is no notable difference between values on the insert and the two grades of COC, indicating that the roughness is replicated with high fidelity on smoother surfaces.

### 3.4. Structure Sealing and Bonding Coverage

**Figure 9:**
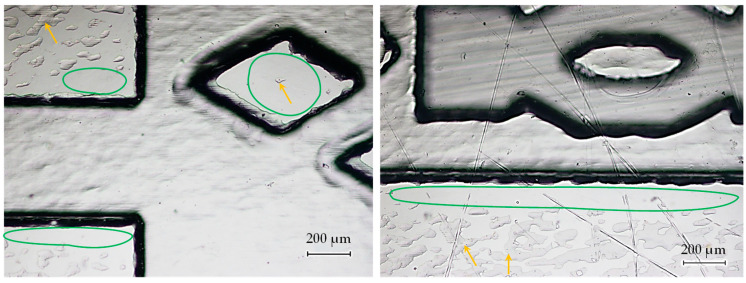
Microscope images of the visual inspection of bonding coverage in two different areas of the benchmark design. The yellow arrows in the images indicate areas with poor bonding coverage, while the areas within the green contour indicate zones with high bonding coverage.

The bonding quality was assessed by visually inspecting the bonding coverage using the optical microscope. For the inspection, the main focus was placed in regions near structure edges and in the rhombus-shaped pillars that are part of the benchmark design. Figure 9 shows that a good bonding coverage was achieved maintaining the integrity of the structures without any damage, as can occur with bonding processes that employ high temperatures and/or pressures or aggressive chemicals for the thermoplastic.

The edges of the structures and the top surface of the pillars were correctly bonded to the cover (marked with green contour in Figure 9). Nonetheless, slightly darker regions in the form of spots have been identified on the upper bonding surface of the larger area (marked with yellow arrows in Figure 9). That variation in the color corresponds to a thin air gap between the injection-molded replica and the cover and is indicative that both mating surfaces have not bonded in that specific areas. The local surface roughness in those small spots prevented the mating surfaces from contact during the process, and therefore those localized regions were not bonded. Even so, the poor bonding coverage in these regions would not affect the correct performance of the fluid flow through the structures, as the edges of the structures were properly sealed. Therefore, it would only be aesthetic defects in the final assembly.

## 4. Conclusions

Femtosecond laser micromachining has shown great potential for a cost-effective fabrication of injection mold inserts for the mass production of microfluidic devices. This work demonstrates the significant potential of fs-laser micromachining for the mass production of functional COC microfluidic devices. In previous works [39], the fabrication of an insert by precision micromilling with an equivalent design was considered. This required 8 h and an approximate cost of EUR 8000 for its fabrication. In this work, approximately 10 times shorter and subsequently 8 times cheaper fabrication times and costs for the production of the insert were needed, compared to when using precision micromilling.

The agility of fs-laser manufacturing enables easily tailoring the production to different chip geometries and sizes, or different processes such as engraving and polishing, without having to employ any process-specific production tool. Following the same strategic line, the use of a reconfigurable master mold of interchangeable inserts during the injection molding permits taking advantage of the flexibility of injection technology.

The replication of microstructures in polymers has been acceptable in general terms, with no notable differences between the two grades of COC, E-140 and 8007S-04. The deviations between the measured dimensions on the polymer parts compared to those of the insert have demonstrated the difficulties in accurately replicating microstructures with relatively high aspect ratios that are located in areas with a high density of structures. This should be a point to consider when designing microfluidic devices. In this work, maximum deviations were registered at the G zone on the benchmark design, having a highest aspect ratio: an error of 39% in the insert compared to the initial designed dimensions, and an error of 19% and 21% for the replicas in COC E-140 and 8007S-04, respectively, compared to the dimensions of the insert were measured.

The fs-laser process developed for microstructure engraving still needs optimization to improve profile rectangularity and remove surface burrs measured at the edges of the microstructures. The roughness in laser-polished surfaces was really smooth on both the insert and the polymer replicas. Sa values of 64.6 nm were measured for the metallic insert and a Sa of 71.8 nm and 72.9 nm for the COC E-140 and 8007S-04 replicas, respectively. However, still, roughness was relatively high at some regions of the COC injection-molded parts, reducing the bonding coverage after the chemical bonding of a cover of the same material on top of the structures to create closed microfluidic channels. The use of a laser beam with a top-hat energy distribution profile would help optimizing the intrinsic issues linked to a Gaussian-shaped laser beam discussed in Section 2.3, without impacting the overall processing time in an important way. Employing shorter laser wavelengths, such as a fs-laser emitting at 515 nm, or an overall lower energy dose would also be beneficial to the quality of the engraved edges but with a very likely increase in the manufacturing time. Nonetheless, the large reduction in manufacturing time observed by laser-based micromachining of the insert with respect to micromilling approach leaves significant room for improvement to achieve better optimization of fs-laser-based micromachining quality.

Additionally, an increase in packing pressure during injection molding of the parts could help to obtain a more complete filling of cavities with high aspect ratios and located in areas with a high density of microstructures, thereby improving replication quality. On the other hand, a higher mold temperature would also contribute to achieving optimized replication. However, the increase in mold temperature tends to prolong the cooling phase, thereby extending the injection cycle duration and increasing the cost per part.

In future lines, it is proposed to study the potential of fs-laser technology for the generation of textured surfaces in COC microfluidic devices. Surface textures have demonstrated the potential to modify the wettability of materials and enhance the cell adhesion and proliferation properties of the surfaces [40,41].

## Figures and Tables

**Table 1:** Final process parameters for structure engraving and polishing by fs-laser.

Process	Repetition Rate (kHz)	Scan Speed (m/s)	Power (W)	Number of Successive Scans	Fluence (J/cm^2^)
Engraving (depth 100 µm)	200	2	18.8	55	12.45
Engraving (depth 200 µm)	200	2	18.8	110	12.45
Polishing	1000 (256 ppb)	1.25	30	2	3.97 (256 ppb total amount)

**Table 2:** Selected injection molding parameters for the fabrication of the replicas in the two different COC grades: E-140 and 8007S-04.

Process Parameters	COC E-140	COC 8007S-04
Melt temperature [°C]	270	220
Mold temperature [°C]	50	50
Injection speed [cm^3^/s]	4	76
Injection volume [cm^3^]	7	4
Packing pressure [bar]	1000	1000
Packing time [s]	6	6
Cooling time [s]	30	30

**Table 3:** Summary of the channel height measurements.

Zone	Designed	Insert		Replica E-140		Replica 8007S-04	
		Measured	Error	Measured	Error	Measured	Error
3-2	100 µm	94.84 µm	5%	87.2 ± 1.2 µm	8%	84.9 ± 1.6 µm	10%
3-2′	100 µm	98.21 µm	2%	87.1 ± 2.2 µm	11%	85.2 ± 1.4 µm	13%
2-1	100 µm	108.9 µm	9%	86.2 ± 1.6 µm	21%	88.2 ± 2.8 µm	19%
2-1′	100 µm	108.8 µm	9%	93.4 ± 4.9 µm	14%	90.8 ± 4.3 µm	17%

**Table 4:** Summary of the channel width measurements.

Zone.	Designed	Insert		Replica E-140		Replica 8007S-04	
		Measured	Error	Measured	Error	Measured	Error
A	750 µm	868.4 µm	16%	855.1 ± 2 µm	2%	864.6 ± 3.9 µm	0%
B	470 µm	552.6 µm	18%	546.5 ± 2 µm	1%	539.9 ± 1.5 µm	2%
C	520 µm	676.6 µm	30%	674.1 ± 2.5 µm	0%	687.0 ± 1.3 µm	2%
D	360 µm	445.1 µm	24%	443.1 ± 2.7 µm	0%	439.5 ± 1.9 µm	1%
E	610 µm	636.6 µm	4%	664.1 ± 2.1 µm	4%	667.1 ± 1.2 µm	5%
F	220 µm	249.6 µm	13%	272.1 ± 0.4 µm	9%	271.6 ± 1.9 µm	9%
G	90 µm	124.8 µm	39%	148.0 ± 2.8 µm	19%	151.3 ± 1.1 µm	21%

## Data Availability

The original contributions presented in this study are included in the article. Further inquiries can be directed to the corresponding author.
